# Antidepressant-like effects of Ashwagandha (Withania Somnifera) on chronic unpredictable mild stress-induced depression in adolescent rats

**DOI:** 10.1007/s00213-025-06844-5

**Published:** 2025-06-25

**Authors:** Gul Sahika Gokdemir, Ugur Seker, Nazan Baksi, Mukadder Baylan, Berjan Demirtaş, Mehmet Tahir Gokdemir

**Affiliations:** 1 Faculty of Medicine, Department of Physiology, Mardin Artuklu University, Mardin, Turkey; 2 Faculty of Medicine, Department of Histology and Embryology, Mardin Artuklu University, Mardin, Turkey; 3 Faculty of Veterinary, Department of Laboratory Animals, Dicle University, Diyarbakır, Turkey; 4 Faculty of Medicine, Department of Physiology, Dicle University, Diyarbakır, Turkey; 5 Vocational School Veterinary Medicine, Plant and Animal Production, Equine and Training Program, Istanbul University-Cerrahpaşa, İstanbul, Turkey; 6 Faculty of Medicine, Emergency Department, Mardin Artuklu University, Mardin, Turkey

**Keywords:** Adolescent depression, Ashwagandha, CUMS, GFAP, Proapoptotic proteins, Sertraline

## Abstract

**Rationale:**

Adolescent depression is often linked to biological changes associated with stress. However, new approaches and treatment strategies for early intervention and prevention of depression in children and adolescents are still limited. Ashwagandha is an Ayurvedic herb widely used in the management of anxiety and stress. However, there is no information in the current literature on its potential effect on adolescent depression.

**Objectives:**

This study aimed to investigate the effects of depression on proapoptotic proteins and neuroinflammation and the antidepressant effect of Ashwagandha on depression-like symptoms in adolescent rats exposed to the Chronic Unpredictable Mild Stress (CUMS) model.

**Methods:**

In the study, CUMS model was used to induce depression in adolescent rats. Rats were treated with Ashwagandha or Sertraline. To evaluate the antidepressant effects, behavioral tests as well as biochemical and histological analyses were performed. Forced Swim Test (FST), Sucrose Test and Elevated Plus Maze Test were performed as behavioral tests. Brain-derived neurotrophic factor (BDNF) and nerve growth factor (NGF) were measured by the ELISA method in the fronto-parietal cortex. Proapoptotic proteins (Bax and Caspase-3) and inflammatory markers (TNF-α and IL-1β), as well as glial fibrillary acidic protein (GFAP), were evaluated immunohistochemically in the fronto-parietal cortex.

**Results:**

Proapoptotic proteins (Bax and Caspase-3) and inflammatory markers (TNF-α and IL-1β) were increased in the CUMS group. BDNF and GFAP levels were decreased. Ashwagandha treatment was more effective than Sertraline in reducing the levels of these proteins and markers. Additionally, Ashwagandha prevented weight loss.

**Conclusions:**

Ashwagandha showed antidepressant-like effects in adolescent rats, reducing apoptosis, inflammation, and neuroinflammation, suggesting potential for treating adolescent depression.

**Graphical Abstract:**

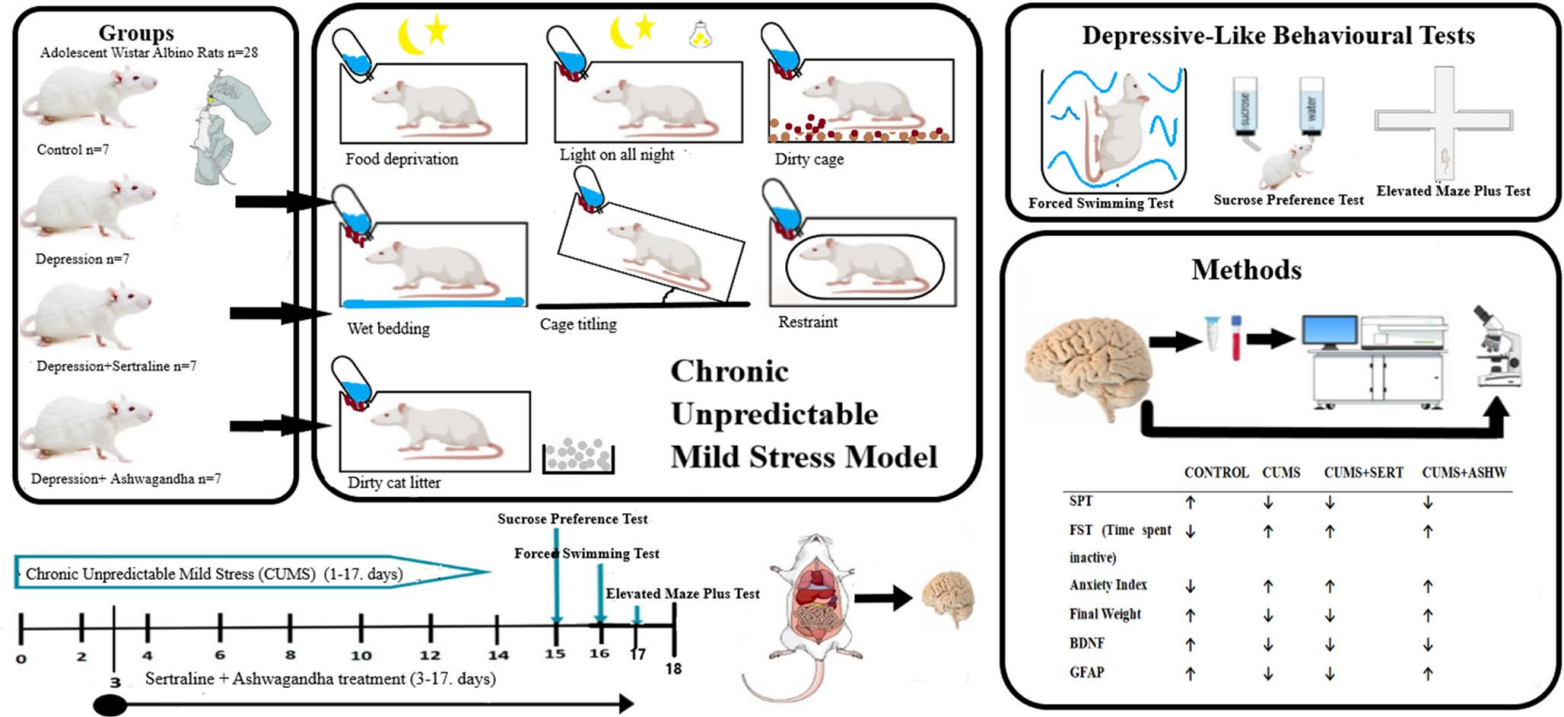

## Introductıon

Depression is becoming increasingly prevalent in adolescents and the general population, representing a significant burden on treatment services (Liu et al. 2024a). Despite extensive research, there are currently no new insights into the prevention and early intervention of depression in children and adolescents.

Depression is widely recognized as a prevalent mental disorder with a complex and multifactorial etiology (Fang et al. 2023). Neurotrophic factors may play important roles in pathophysiology of depression, as they regulate neuronal plasticity, especially within the hippocampus and prefrontal cortex. Recent studies suggest that alterations in neurotrophic factors, particularly reduced levels of brain-derived neurotrophic factor (BDNF), may be associated with the development of depression (Porter and O’Connor 2022; Wiener et al. 2015). However, studies on the nerve growth factor (NGF), another crucial neurotrophic factor, have yielded contradictory results (Wiener et al. 2015).

The role of inflammation in the aetiology of depression is also becoming increasingly significant. Elevated levels of inflammatory cytokines have been observed in individuals with depressive disorders, and it has been implicated that these cytokines may exacerbate depressive symptoms by promoting neuroinflammation (Liu et al. 2024b). Glial fibrillary acidic protein (GFAP) is a specific biomarker protein expressed by astrocytes. Growing evidence suggests that GFAP is predominantly associated with a range of neurological injuries, inflammatory processes and nervous system disorders (Zheng et al. 2024). Furthermore, the Bax and caspase-3 proteins, key mediators of apoptosis,, have been demonstrated to be elevated in individuals with depression (Dionisie et al. 2021). In animal studies, it has been demonstrated that chronic stress increases caspase-3 activity, decreases Bcl-2 levels, and promotes neuronal apoptosis by decreasing neurotrophic factors such as BDNF (Dionisie et al. 2021). These findings suggest that chronic stress may trigger pro-apoptotic mechanisms that accelerate cell death and contribute to the development of depressive behaviors through both the acceleration of cell death and inhibition of neurogenesis.

Serotonin, a selective serotonin reuptake inhibitor (SSRI), is a group of antidepressants commonly used to treat depression in adolescents.(Pereira-Figueiredo et al. 2014). In addition, Withania somnifera, known as ‘Ashwagandha’ in traditional Indian medicine, has been shown to stimulate the immune system, prevent inflammation and support neuronal plasticity (Mukherjee et al. 2021). However, to the best of our knowledge, there is no study about the effect of ashwagandha on adolescent depression.

The fronto-parietal cortex plays an important role in executive functions and emotional regulation and is central to social cognition (Wurm and Caramazza 2019). Neuroimaging studies reveal that depressed patients exhibit alterations in cognitive function circuits compared to healthy controls. In particular, it has been reported that structural or functional changes in the fronto-parietal cortex may be associated with depressive symptoms (Schultz et al. 2018).

This study aimed to investigate the antidepressant-like effects of Ashwagandha on chronic unpredictable mild stress induced depression in adolescent rats. The hypothesis is that Ashwagandha alleviates the depresion like behaviours through the modulation of BDNF and NGF levels and decreasing the proapoptotic Bax and caspase-3 activation, tumour necrosis factor-alpha (TNF-α) and interleukin-1β (IL-1β) cytokines and increasing GFAB expression. In this context, in our study, immunohistochemistry and biochemical analyses were performed in the frontal-parietal cortex and the changes in this region were examined.

## Materıals and methods

### Experimental animals

Adolescence in rodents occurs between postnatal days 35 and 49, a period during which there is an enhancement in behavioral and cognitive control capabilities (Semple et al. 2013; Blakemore 2008). Thus, our study included 28 adolescent male Wistar albino rats, aged 35–49 days and weighing between 150 and 180 g. Animal care was performed in accordance with the guidelines for experimental animals during the process and experimental phase. The animal experiments were performed in accordance with the local ethics committee of Dicle University (Ethical Approval No: 2023/14 Date: 03/05/2023).

### Experimental design

Animals in all groups were fed with standard normal diet and tap water in stainless steel cages.

The rats were randomly divided into four groups, each consisting of seven rats as follows;

1. Control
2. Chronic Unpredictable Mild Stress induced (CUMS)
3. CUMS treated with Sertraline (CUMS + SERT)
4. CUMS treated with Ashwagandha (CUMS + ASHW)

Control group animals were fed with normal diet and tap water at 22 ± 2 ºC for 12 h in light and 12 h in darkness without any restriction. The rats in this group were given 0.5 cc saline by oral gavage from the 3rd and 17th day. In CUMS group, CUMS model was applied and 0.5 cc saline was given by oral gavage 1 h before exposure to stressors, from the 3rd to the 17th day. In CUMS + SERT group, CUMS model was applied and sertraline dissolved in water (Selectra 25 mg Film Coated Tablet) at a dose of 5.0 mg/kg/day was given by oral gavage 1 h before exposure to stressors, from the 3rd day to the 17th day (Pereira-Figueiredo et al. 2014). In CUMS + ASHW group, CUMS model was applied and Ashwagandha (Powdered ashwagandha extract Sigma-Aldrich Chemie GmbH) dissolved in water at a dose of 50 mg/kg/day was given by oral gavage 1 h before exposure to stressors, from the 3rd to the 17th day (Bhattacharya et al. 2001).

### Chronic unpredictable mild stress model (CUMS)

Rats in the experimental groups (CUMS, CUMS + SERT, CUMS + ASHW) were exposed to chronic stressors in a semi-randomised manner and at specific time intervals (Willner 2017; Koc and Sahin 2018). These stressors included 7 h in a wet cage, overnight starvation, 10 min exposure to cat litter, 45 min physical restraint, continuous lighting overnight, 7 h in an inclined cage, and 7 h in a dirty cage (Table 1). These treatments started from the 1 st day of the experiment and continued until the 17th day. Rats in the control group were kept in a separate room without contact with stressed rats. They were not disturbed except for necessary routine procedures such as cage cleaning.

**Table 1:** Experimental scheme of applied chronic unpredictable mild stress model

	Behavioral Test	Morning			Noon			Night		
Day		Hour	Stressor	Duration						
1		09.00	Wet bedding	7 h				16.00–08.00	Food deprivation	All night
2		10:00	Dirty cat litter	10 min	15:00	Restraint	45 min	16.00–08.00	Light on	All night
3		09:00	Cage titling	7 h						
4		11:00	Restraint	45 min	14:00	Dirty cat litter	10 min			
5		10:00	Wet bedding	7 h				16.00–08.00	Food deprivation	All night
6		09:00	Dirty cat litter	10 min	15:00	Restraint	45 min			
7		11:00	Dirty cage	7 h				16.00–08.00	Light on	All night
8		09.00	Wet bedding	7 h				16.00–08.00	Food deprivation	All night
9		10:00	Dirty cat litter	10 min	15:00	Restraint	45 min	16.00–08.00	Light on	All night
10		09:00	Wet bedding	7 h						
11		11:00	Restraint	45 min	14:00	Dirty cat litter	10 min			
12		10:00	Cage titling	7 h				16.00–08.00	Food deprivation	All night
13	SPT	09:00	Restraint	45 min						
14		11:00	Dirty cage	7 h				16.00–08.00	Light on	All night
15	SPT	09.00	Wet bedding	7 h				16.00–08.00	Food deprivation	All night
16	FST 09:00–2:00	10:00	Dirty cat litter	10 min	15:00	Restraint	45 min	16.00–08.00	Light on	All night
17	EPM 09:00–2:00	09:00	Cage titling	7 h						

### Depressive-like behavioural tests

Sucrose Preference Test (SPT), Forced Swim Test (FST) and Elevated Plus Maze Test (EPM) tests were applied to all groups to determine depression-like behavioural features. Double blindness was used for behavioural tests. Behavioral tests were scored manually by three investigators. To minimize bias, all investigators were blinded to treatment groups during analysis. All behavioural tests were performed between 9:00 a.m. and 2:00 p.m. In addition, all rats were assessed after a 15-minute habituation period in the test room.

### Sucrose preference test (SPT)

SPT was performed on the 15th day of the experiment to measure anhedonia in rats. On the 13th day of the experiment, two different water bottles, each weighing 580 g, were placed on the right and left sides of the cage. The experimental animals were allowed to drink water from both bottles for 24 h. After 2 days of training, water containing 2% sucrose was randomly placed in one of the bottles. The bottles were weighed before and after 24 h. Water and sucrose consumption was evaluated as total consumption (Santiago et al. 2015). The decrease in the consumption of pleasant liquids and food was evaluated as anhedonia (Castagne et al. 2009; Valvassori et al. 2013).

Sucrose Preference (%) = Sucrose consumption x100/total consumption (Santiago et al. 2015).

### Forced swimming test (FST)

FST was performed on the 16th day of the experiment. A 40 cm high and 25 cm in diameter glass cylinder was filled to a depth of 30 cm with water at 20–25 °C (Taşkıran et al. 2024). The water was changed for each animal before the test. For the FST protocol, FST was applied to the rats for acclimatisation purposes for 15 min on the 14th day of the experiment. After the experiment, they were dried and placed back in their cages. On the experimental day, rats were left in a glass cylinder filled with water for FST for 5 min (Koc and Sahin 2018). Two different parameters, immobility and swimming, were evaluated. Swimming and immobilisation times of the rats were determined by camera recordings. Immobility was defined as the behavior in which the animal remains motionless while keeping its head above the water, without any effort or escape movements. Swimming, on the other hand, was defined as the behavior in which the animal actively swam and made efforts to struggle and escape.

### Elevated plus maze test (EPM)

EPM test was performed on the 17th day of the experiment to measure anxiety in rats. Labyrinths of 50 cm in height and 10 cm in width, containing open and closed spaces, are used to examine the recognition behaviour of rats in unfamiliar environments (Aykaç et al. 2015). Considering that rats are innately afraid of open and high places in EPM experiments, rats are usually left in the centre with their faces towards one of the open arms (Aykaç et al. 2015). The indicators used to evaluate anxiety include an increased time spent in the closed arm, prolonged freezing time, a reduced number of entries into the open arm, decreased time spent in the central area, and increased frequency and duration of standing on two legs and sniffing the air (El-Kadi et al. 2024). After each test, all areas traversed by the subjects were cleaned with 70% ethanol. Each rat was placed on the central platform, and its movements within the Elevated Plus Maze (EPM) were recorded for a duration of 5 min.

The anxiety index was subsequently calculated using the following equation:

Anxiety index = 1-([time spend in open arms/5 minutes] + [number of entries to the open arms/total exploration to the maze])/2 (El-Kadi et al. 2024).

Values close to 1 indicate a higher level of anxiety (El-Kadi et al. 2024).

### Sample collection

The weights of the rats were weighed before and at the end of the experiment. On the 18th day of the experiment, following 12 h-fasting period, all animals were sacrificed by cardiac puncture under anaesthesia with 10 mg/kg xylazine and 90 mg/kg ketamine HCl injection. Brain tissue samples were collected from the right and left fronto-parietal cortex separately. The right fronto-parietal cortex tissue was stored at −80 °C until enzyme-linked immunosorbent assay (ELISA) analysis. For further analysis of histopathological and immunohistochemical measurements, samples from the left fronto-parietal cortex tissue were fixed in 10% neutral formalin.

### Biochemical analysis

For BDNF and NGF analysis, homogenates were prepared from right fronto-parietal tissue stored at −80 °C. Brain tissues were removed from the freezer, placed in a glass tube containing phosphate buffer solution (PBS) at 1:10 (w/v, pH:7.2) and homogenised on ice with a tissue homogeniser (Bandelin, UW 2070, Sigma, St. Louis, MO) at 15,000 rpm for 60 s. Homogenates were placed in centrifuge tubes and centrifuged at 5000 rpm for 10 min at 4 °C. The supernatants were transferred to an eppendorf tube. BDNF and NGF levels in tissue supernatants were read spectrophotometrically at 450 nm on a microplate reader (Biochrom, Anthos Zenyth 200) using special ELISA kits (Sun Red, cat. no: 201-11-0477, Sun Red, cat. no: 201-11-0540) according to the manufacturer’s instructions.

### Tissue processing and routine staining

The fronto-parietal cortex and white matter of the brain tissue were examined under light microscope. For this purpose, samples taken from the left cerebral hemispheres were fixed in 10% formalin solution and routine tissue follow-up was performed for histopathological and immunohistochemical examination (Sagir et al. 2023). The fixed tissues were washed under running tap water and dehydrated in a series of increasing grade alcohols and then cleared in xylene solution. Paraffin infiltrated samples were then embedded in paraffin blocks. The 5 μm cross sections were taken from the tissue paraffin blocks and received on charged slides for staining. For routine pathological examination, all prepared sections were stained with haematoxylin-eosin (HE) and micrographs were received.

### Immunohistochemical procedures

Pro-apoptotic Bax and Caspase 3, pro-inflammatory TNF-α and IL-1β immunoexpressions and astrocyte-specific GFAP immunohistochemical staining protocol were performed in the obtained sections (Dicle et al. 2023). For this purpose, the sections were deparaffinised in xylene and rehydrated in decreasing alcohol series. The sections were then heated in citrate buffer (pH: 6.0) and antigen retrieval was performed. Tissue samples were incubated in 3%H2O2 for endogenous peroxidase inhibition and blocking solution (Thermo Scientific, Waltham, MA, USA, Catalog number: TA-125-HL) was used for inhibition of non-specific binding. Tissue samples were diluted 1:100, 1:100, 1:300, 1:100 and 1:100 respectively with Bax (Santa Cruz Biotechnology (SCBT), DTX, MA, USA, Catalog number: sc-7480) Caspase 3 (Santa cruz technology, MA, USA, Catalog number: sc-56053), TNF-α (Santa Cruz Biotechnology (SCBT), DTX, MA, USA, Catalog number: sc-52746), IL-1β (Santa Cruz Biotechnology (SCBT), DTX, MA, USA, Catalog number: sc-52012) and GFAP (Santa Cruz Biotechnology (SCBT), DTX, MA, USA, Catalog number: sc-33673) overnight at 4 °C. Subsequently, the samples were washed in PBS and immunodetection was performed using a kit containing secondary antibody and enzyme (Thermo Scientific, Waltham, MA, USA, Catalog number: TP-125-HL). A ready-to-use DAB chromogen kit (Thermo Scientific, Waltham, MA, USA, Catalog number: TA-125-HD) was used for detection of antibodies and the kit was applied according to the manufacturer’s instructions. The reacted samples were counterstained with haematoxylin, covered with entellan and examined under a light microscope and micrographs were taken.

### Measurement of immunodensity and GFAP positive cell index

Bax, Caspase 3, TNF-α and IL-1β immunoexpressions in immunohistochemistry stained samples from the fronto-parietal cortex were analysed in terms of density by threshold analysis (Gokdemir et al. 2024). For this purpose, threshold analysis was performed with ImageJ software by proportioning the DAB positive brown area to the total cross-sectional area of the tissue in 4 different areas in the fronto-parietal cortex tissue of each animal. The analyses were manually converted to percentage values and the percentage data obtained were statistically analysed. Astrocyte cells were counted by counting the number of specific GFAP positive cells, and the cell density level was obtained by averaging the GFAP positive cells in 4 different randomly selected areas in the white matter in the fronto-parietal cortex section of each animal at large magnification to the total number of cells and converting the obtained values into percentages to obtain the percentage of GFAP positive cells. All immunodensity and positive cell index measurements were evaluated statistically.

### Statistical analysis

Statistical evaluation was performed using SPSS 26.0 (SPSS Inc., Chicago, IL, USA). Normal distribution was assessed using the Shapiro-Wilk test, and arithmetic mean ± standard deviation was used for continuous variables with normal distribution, while frequency and percentage were used for categorical variables. One-Way ANOVA was applied for comparison of continuous data, and Tukey HSD post-hoc test was used for intra-group comparisons. Chi-square test was used for categorical variables. In all evaluations, *p* < 0.05 and *p* < 0.01 were considered as statistically significant.

## Results

### SPT

**Fig. 1:**
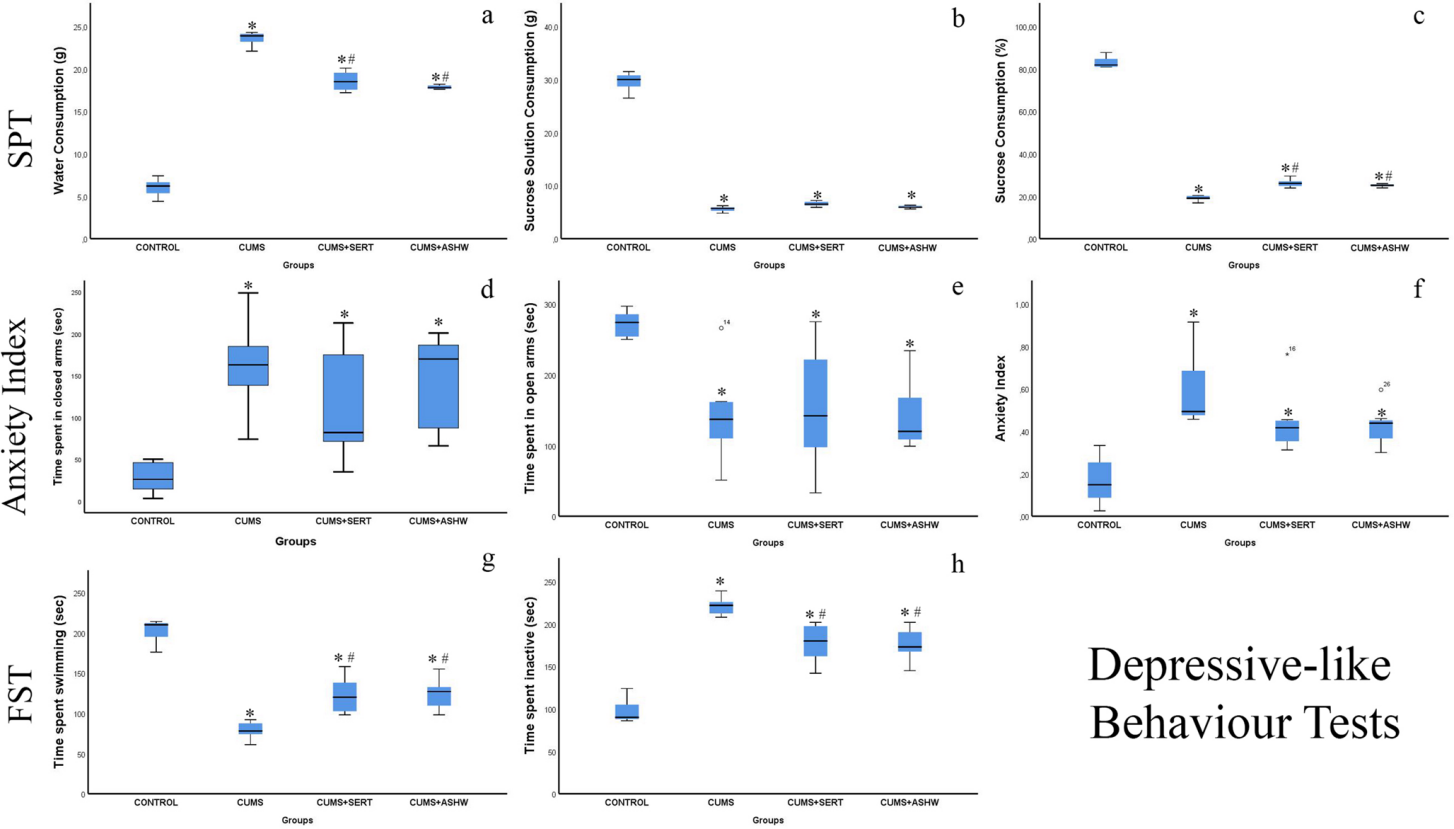
(**a**–**c**) SPT parameters: water consumption, sucrose solution consumption, and sucrose consumption percentage. (**d**–**f**) Elevated Plus Maze parameters: time spent in closed arms, time spent in open arms, and anxiety index. (**g**–**h**) FST parameters: time spent swimming and time spent inactive. Each bar with vertical line represents mean ± S.D. of 7 rats per group. Statistical significance was determined using one-way ANOVA followed by Tukey’s multiple comparison test. Significance is indicated as follows:*P < 0.05 vs. CONTROL, #*P* < 0.05 vs. CUMS. SPT: Sucrose Preference Test; FST: Forced Swimming Test; CUMS: Chronic Unpredictable Mild Stress induced rats; CUMS + SERT: Chronic Unpredictable Mild Stress was treated with Sertraline; CUMS + ASHW: Chronic Unpredictable Mild Stress was treated with Ashwagandha

Total water consumption, sucrose solution consumption and sucrose consumption percentages for each group are summarized in Table 2. Significant differences were found between the groups in terms of water consumption, sucrose solution consumption and sucrose consumption percentage (F(3,24) = 1013.363, *p* < 0.001; F(3,24) = 450.007, *p* < 0.001; F(3,24) = 1832.777, *p* < 0.001, respectively). The control group showed significantly higher sucrose solution consumption (207 g, total consumption of 7 rats) and a sucrose consumption percentage rate of 83.14 ± 2.78% compared to the other groups. The sucrose solution consumption percentage was significantly lower in the CUMS group (39 g, 19.10 ± 1.25%, *p* < 0.001). Sucrose consumption percentages in CUMS + SERT and CUMS + ASHW groups were found to be significantly higher than in CUMS group (26.17 ± 1.99% and 25.14 ± 0.72%, respectively, *p* < 0.001). Sucrose consumption percentages remained similar in both treatment groups (*p* = 0.733). Water consumption increased in CUMS group compared to the control group (23.57 ± 0.84, *p* < 0.001), but decreased in CUMS + SERT (18.57 ± 1.21) and CUMS + ASHW (17.86 ± 0.24) groups compared to the CUMS group (*p* < 0.001). Sucrose solution consumption was determined to be the highest in the control group (29.57 ± 1.81 g) and the lowest in the CUMS group (5.57 ± 0.51 g). Consumption in the CUMS + SERT and CUMS + ASHW groups was higher than that in the CUMS group (6.57 ± 0.49 g and 6.00 ± 0.25 g, respectively, *p* < 0.001). These findings indicate the ameliorative effect of Ashwagandha and sertraline on the decreased sucrose consumption due to the CUMS model (Fig. 1).

**Table 2:** Effects of stress, Ashwagandha and Sertraline treatments on sucrose preference test

	Sucrose Consumption (%) Mean ± SD	Water Consumption (g) Mean ± SD	Sucrose Solution Consumption (g) Mean ± SD	Total Water Consumption (g)	Total Sucrose Solution Consumption (g)
CONTROL	83.14 ± 2,78	6.00 ± 1.10	29.57 ± 1.81	42	207
CUMS	19.10 ± 1.25^a^	23.57 ± 0.84^a^	5.57 ± 0.51^a^	165	39
CUMS + SERT	26,17 ± 1.99^a, b^	18.57 ± 1.21^a, b^	6.57 ± 0.49^a^	130	46
CUMS + ASHW	25.14 ± 0.72^a, b^	17.86 ± 0.24^a, b^	6.00 ± 0.25^a^	125	42

### FST

The immobilisation time and swimming times of the groups in the FST are presented in Table 3. The analysis results regarding immobility duration revealed a significant difference between the groups (F (3, 24) = 55.960, *p* < 0.001). Similarly, swimming times also showed a statistically significant difference between the groups (F (3, 24) = 55.960, *p* < 0.001). The results demonstrated that stress significantly increased the immobility time and significantly decreased the swimming times in the CUMS group compared to the control group (*p* < 0.001). Additionally, significant differences in immobility time and swimming times were observed between the CUMS group and the SERT and ASHW treated depression groups (for both; *p* < 0.001). However, no significant differences in immobility time and swimming times were found between the CUMS + SERT and CUMS + ASHW groups (*p* > 0.05) (Figüre 1).

**Table 3:** Behavioral test parameters of the groups

Parameters	Control	CUMS	CUMS + SERT	CUMS + ASHW
FST (Time spent inactive, sec)	98.00 ± 16.50^a^	220.86 ± 10.96^c^	177.57 ± 22.92^b^	176.57 ± 19.82^b^
FST (Time spent swimming, sec)	202.00 ± 16.50^a^	79.14 ± 10.96^c^	122.43 ± 22.92^b^	123.43 ± 19.82^b^
Anxiety Index	0.17 ± 0.12^a^	0.59 ± 0.17^d, e^	0.44 ± 0.15^d, e^	0.42 ± 0.10^d^

### Anxiety index

Analysis results regarding anxiety index showed that there was a statistically significant difference between the groups (F(3, 24) = 11.593, *p* < 0.001). This finding reveals that the anxiety index differed between the treatment groups. The CUMS group showed an increased anxiety index compared to the control group. Rats in the CUMS group exhibited increased time in the closed arm and decreased time in the open arm, and this difference was statistically significant (*p* < 0.01) (Fig. 1). Although both SERT and ASHW treatments resulted in a decreased anxiety index compared to the CUMS group, this decrease was not statistically significant (*p* > 0.05). Furthermore, no statistically significant difference was found between the SERT and ASHW treatment groups (*p* > 0.05, Table 3).

Typical depression phenotypes after CUMS include decreased body weight gain, sucrose preference, and increased immobility time in the forced swim test (Qiao et al. 2014). Our data revealed that all CUMS groups showed typical depressive-like behaviors, such as lower sucrose preference, higher immobility time on the FST, and increased anxiety index, compared with the control group.

### Weight change

**Fig. 2:**
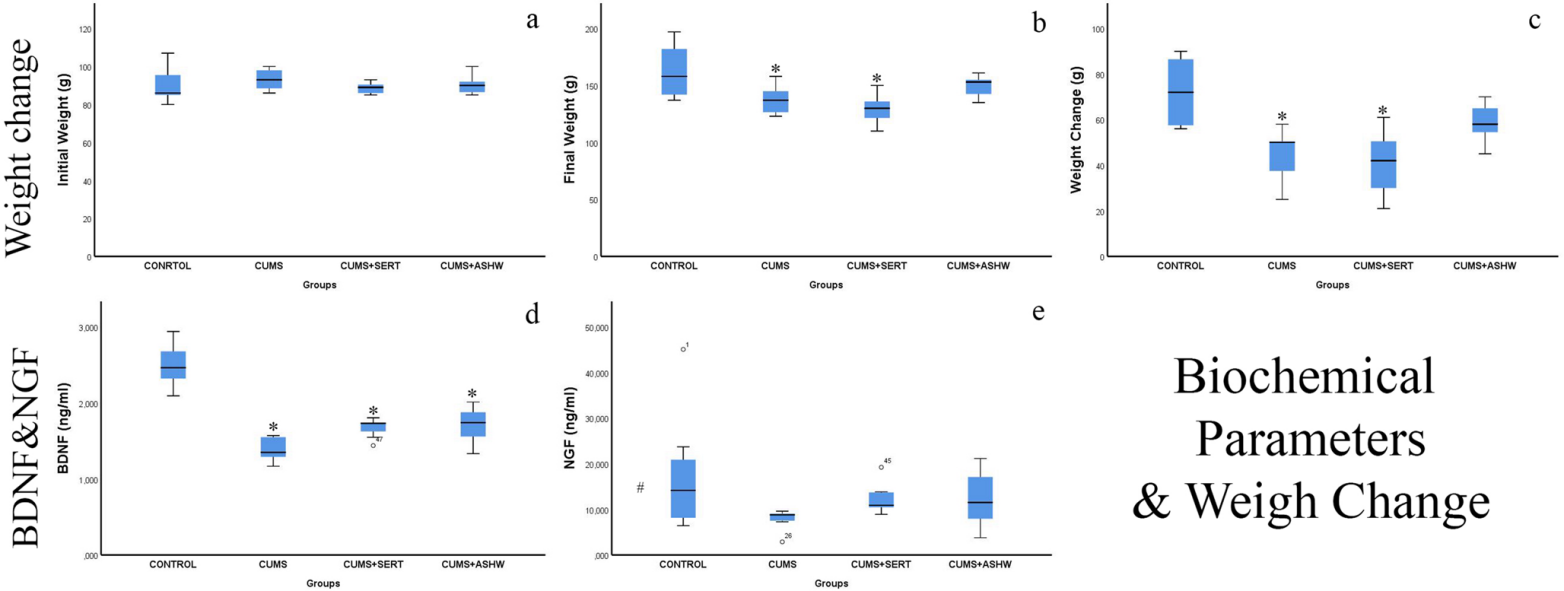
Histopathological micrographs of the fronto-parietal cortex and white mater in Control, CUMS, CUMS + SERT and CUMS + ASHW groups. Cortical neurons (arrow), and glial cells (curved arrow) with pyknotic nuclei. Existence of increased pericellular cavity due to presence of edema in white mater glial cells (curved arrow). The increased perivascular cavity (arrowhead) and accumulation of edema in stratum moleculare (asterisk) which is the most upper layer of the fronto-parietal cortex. The observed pathological changes are partially alleviated in CUMS + SERT group but the edema was still obvious in perivascular cavity (arrowhead). The neuronal and glial cell morphology and edema in cortex and white mater vessels (thick arrow) is remarkably reduced in CUMS + ASHW group. CUMS: Chronic Unpredictable Mild Stress induced rats; CUMS + SERT: Chronic Unpredictable Mild Stress was treated with Sertraline; CUMS + ASHW: Chronic Unpredictable Mild Stress was treated with Ashwagandha

There was no significant difference between the groups in terms of initial weights (F(3, 24) = 0.554, *p* = 0.651). However, there were significant differences between the groups in final weights (F(3, 24) = 5.728, *p* = 0.004) and weight changes (F(3, 24) = 8.780, *p* < 0.001). CUMS (44.14 ± 11.276) and CUMS + SERT group (40.71 ± 15.174) showed a statistically significant decrease in weight compared to the control group (*p* = 0.002 and *p* = 0.001, respectively).There was no significant difference in weight change between the control group and the ASHW-treated group (*p* > 0.05) (Table 4; Fig. 2). These findings indicate that the treatment groups had a notable impact on both final weights and weight changes.

**Table 4:** Weight change in the groups

PARAMETERS	CONTROL	CUMS	CUMS + SERT	CUMS + ASHW
Initial Weight(g)	90.57 ± 10.49^a^	93.14 ± 5.79^a^	88.57 ± 3.10^a^	90.29 ± 5.19^a^
Final Weight (g)	162.86 ± 24.48^b^	137.29 ± 12.89^a^	129.29 ± 13.96^a^	149.14 ± 9.25^a, b^
Weight Change (g)	72.29 ± 15.39^a^	44.14 ± 11.28^c^	40.71 ± 15.17^c^	58.86 ± 8.78^a, c^

### Biochemical analyses

A statistically significant difference was found in BDNF levels between the groups (F(3, 24) = 33.194, *p* < 0.001). BDNF levels were found to be significantly decreased in the CUMS group (1.397 ± 0.164 ng/mL) compared to the control group (2.501 ± 0.291 ng/mL) (*p* = 0.000). No significant increase was observed in the BDNF levels of the treatment groups (SERT: 1.674 ± 0.130 ng/mL, ASHW: 1.709 ± 0.249 ng/mL) compared to the CUMS group (*p* > 0.05). However, the difference in the CUMS + ASHW group compared to the CUMS group was borderline (*p* = 0.059). In addition, no significant difference was found between the BDNF levels of the SERT and ASHW treated groups (*p* > 0.05). No statistically significant difference was found in NGF levels between the groups (F(3, 24) = 1.891, *p* = 0.158). Consistent with this finding, Tukey HSD post-hoc analysis also showed no significant differences between groups (*p* > 0.05). (Table 5; Fig. 2).

**Table 5:** BDNF and NGF levels in control and experimental groups

Parameters	Control	CUMS	CUMS + SERT	CUMS + ASHW
BDNF (ng/mL)	2.50 ± 0.29^a^	1.40 ± 0.16^b^	1.67 ± 0.13^b^	1.71 ± 0.25^b^
NGF (ng/mL)	17.68 ± 13.60^a^	7.78 ± 2.31^a^	12.49 ± 3.47^a^	12.37 ± 6.33^a^

### Histopathological observations

**Fig. 3:**
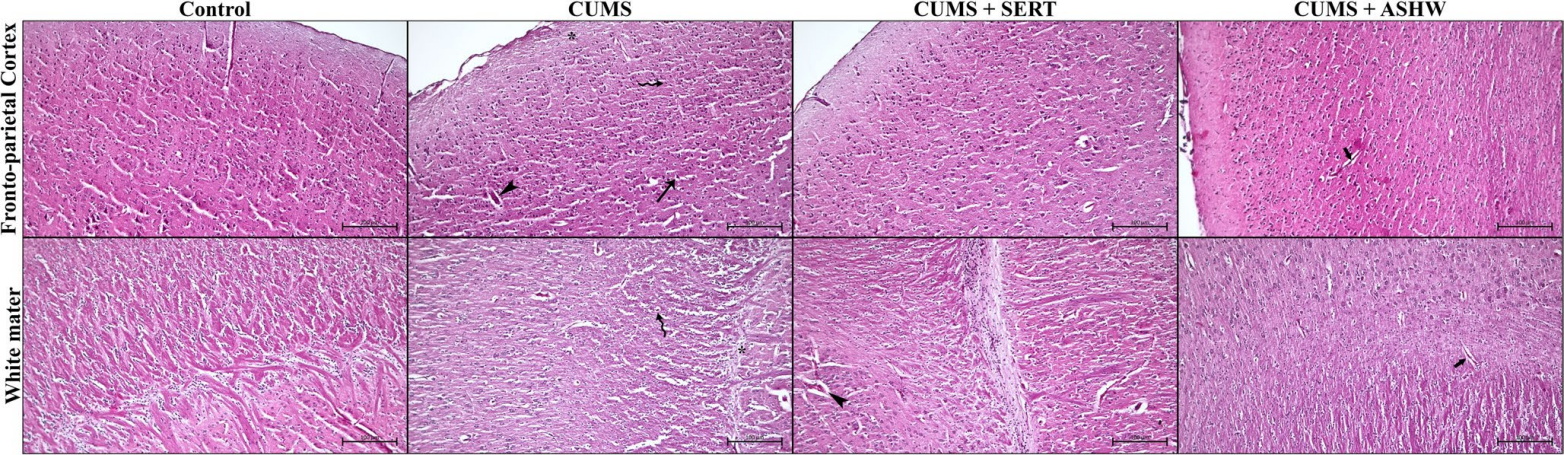
Histopathological micrographs of the fronto-parietalcortex and white mater in Control, CUMS, CUMS + SERT and CUMS + ASHW groups. Cortical neurons (arrow),and glial cells (curved arrow) with pyknotic nuclei. Existence of increased pericellular cavity due to presence of edema in white mater glial cells (curved arrow). The increased perivascular cavity (arrowhead) and accumulation of edema in stratum moleculare (asterisk) which is the most upper layer of the fronto-parietal cortex. The observed pathological changes are partially alleviated in CUMS + SERT group but the edema was still obvious in perivascular cavity (arrowhead). The neuronal and glial cell morphology and edema in cortex and white mater vessels (thick arrow) is remarkably reduced in CUMS + ASHW group.CUMS: Chronic Unpredictable Mild Stress induced rats; CUMS+SERT: Chronic Unpredictable Mild Stress was treated with Sertraline; CUMS+ASHW: Chronic Unpredictable Mild Stress was treated with Ashwagandha

Our examinations indicated that fronto-parietal cortex structure in Control group was regular. Cortex was filled with nerve fibers, various shaped neuron and glial cells. The white mater of the control brain was structured with collaterally branched nerve fibers and glial cells (Fig. 3). The fronto-parietal cortex structure in CUMS group was similar both in cortical and white mater in terms of histological observations. However, in this group we observed increase in perivascular and perineural cavity which is possible a result of accumulation of edema in cerebral structure. Some of the glial cells and neuron were observed with pyknotic nuclei, but the morphological pathology in this group was not severe. When the morphological structure in CUMS + SERT and CUMS + ASHW, the fronto-parietal cortex and white mater in these groups were filled with central nervous system structures. The cortical neurons and glial cells were regular and both of the myelinated and unmyelinated nerve fibers were visible exactly. The white mater of these groups were more similar to the Control group in contrast to CUMS group. The nerve fiber branches were more regular and neuroglia cells were more close to the Control group when the cellular morphology is considered.

### Immunohistochemistry results

**Fig. 4:**
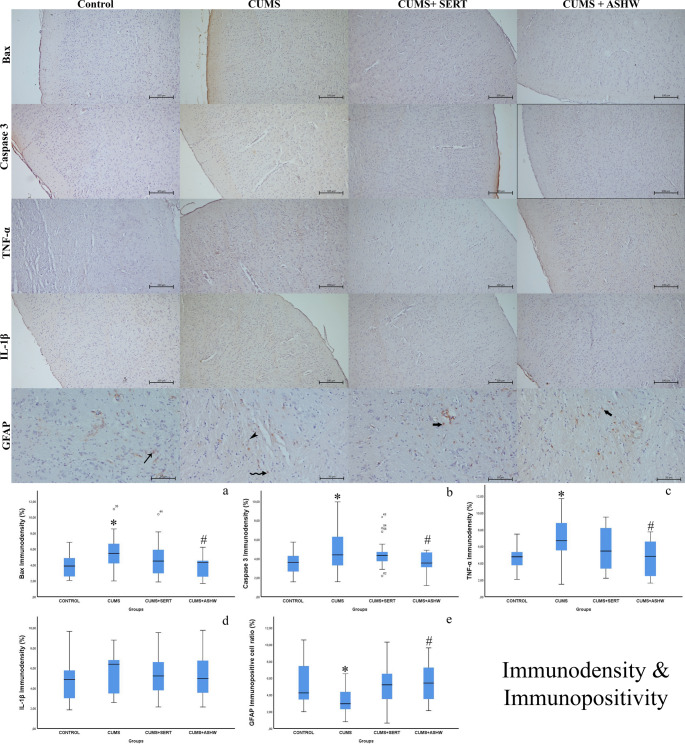
Demonstrative immunohistochemistry micrographs of the Bax, Caspase 3, TNF-α and IL-1β in Control, CUMS, CUMS + SERT and CUMS + ASHW groups. Existence of the brown color indicate immunopositivity of the interest protein. The samples are counterstaind to determine the immunodensity in each group. Counterstain: Hematoxylin. Bar: 100 µm.Representative micrographs of the GFAP immunohistochemistry in Control, CUMS, CUMS + SERT, CUMS + ASHW groups. Regular immunopositivity in astrocytes in control group (arrow). The immunodensity (curved arrow) and distribution of the immunpositive cells (arrow head) ratio is significantly reduced in CUMS group. The GFAP positive cells (thick arrow) in CUMS + SERT and the CUMS + ASHW groups. Counterstain: Hematoxylin. Bar: 50 µm. **a**: Bax, **b**: Caspase-3, **c**: TNF-α, **d**: IL-1β, **e**: GFAP. Each bar with vertical line represents mean ± S.D. of 7 rats per group. Statistical significance was determined using one-way ANOVA followed by Tukey’s multiple comparison test. Significance is indicated as follows: *P < 0.05 vs. CONTROL, #*P* < 0.05 vs. CUMS. CUMS: Chronic Unpredictable Mild Stress induced rats; CUMS+SERT: Chronic Unpredictable Mild Stress was treated with Sertraline; CUMS+ASHW: Chronic Unpredictable Mild Stress was treated with Ashwagandha

When the pro-apoptotic Bax and Casapase 3, pro-inflammatory TNF-α and IL-1β were considered, the cells and nerve tissue were positive for all of these cellular proteins in varying levels (Fig. 4). The glial cell specific GFAP is mainly expressed in the white mater of the cerebrum. The cortical neurons, both cortical and white matter glia cells were containing in a very low amount of pro-apoptotic Bax and Caspase-3. There were significant differences between the groups in terms of Bax levels (F(3,80) = 4.374, *p* = 0.007), Caspase-3 levels (F(3,80) = 3.834, *p* = 0.013), TNF-α levels (F(3,80) = 4.523, *p* = 0.006) and GFAP levels (F(3,80) = 4.236, *p* = 0.008). However, no significant difference was found in terms of IL-1β levels (F(3,80) = 0.651, *p* = 0.585). When the statistical results of the pro apoptotic Bax and Caspase-3 were evaluated, there were significantly difference between Contol and CUMS group (*p* < 0.05). In addition, the pro-inflammatory TNF-α and IL-1β level in CUMS group is upregulated significantly (*p* < 0.05), but the astrocyte specific GFAP positive cell ratio is significantly reduced (*p* < 0.05) compared to Control group. When the immunodensity of pro-apoptotic Bax and Caspase 3, pro-inflammatory TNF-α and IL-1β, and astrocytic GFAP positive cell ratios were considered in CUMS + SERT group, the immunodensity levels in this group was similar (*p* > 0.05) to the both Control and CUMS groups. In addition, the pro-apoptotic Bax and Caspase levels in CUMS + ASHW was similar (*p* > 0.05) to the Control and CUMS + SERT groups, but the levels of these apoptotic proteins are dramatically reduced (*p* < 0.05) in this group compared to CUMS group. The inflammatory cytokine of TNF-α in CUMS + ASHW was significantly different (*p* < 0.05) than CUMS group, but the level of IL-1β in this group was similar (*p* > 0.05) to the rest of the groups. When the GFAP positive cell ratios were examined the positive astrocyte ratio of the CUMS + ASHW was similar (*p* > 0.05) to the both Control and CUMS + SERT groups, but there was a significantly difference (*p* < 0.05) between CUMS and the CUMS + ASHW group. The detailed statistical analysis of the immunodensity examinations are shown in Table 6.

**Table 6:** Statististical analysis results of bax, caspase 3, TNF-α and IL-1β immunodensity, and GFAP immunopositive cell ratios in control, CUMS, CUMS + SERT and CUMS + ASHW groups

	Bax immunodensity (%)	Caspase 3 immunodensity (%)	TNF-α immunodensity (%)	IL-1β immunodensity (%)	GFAP immunopositive cell ratio (%)
CONTROL	3.84 ± 1.38^a^	3.52 ± 1.15^a^	4.69 ± 1.47^a^	4.73 ± 1.99^a^	5.22 ± 1.99^a^
CUMS	5.57 ± 2.16^b^	4.88 ± 2.32^b^	6.82 ± 2.49^b^	5.57 ± 1.95^a^	3.22 ± 1.95^b^
CUMS + SERT	4.72 ± 2.15^a, b^	4.49 ± 1.48^a, b^	5.74 ± 2.56^a, b^	5.28 ± 1.97^a^	5.00 ± 1.97^a, b^
CUMS + ASHW	3.84 ± 1.42^a^	3.51 ± 1.27^a^	4.64 ± 2.18^a^	5.38 ± 2.23^a^	5.51 ± 2.23^a^

## Discussion

In our study, the antidepressant effect of Ashwagandha in adolescent rats was investigated in CUMS model of depression. Withania somnifera, known as Ashwagandha, is an ayurvedic herb used in the treatment of anxiety and stress, which is accepted as an adaptogen showing the ability to stabilise the body’s response to stress by regulating physiological processes (Provino 2010). Studies have shown that Ashwagandha extract alleviates stress symptoms and enhances stress tolerance in rats exposed to chronic stress (Bhattacharya and Muruganandam 2003). Additionally, it has been demonstrated that Ashwagandha produces similar effects to lorazepam in rats. These findings suggest that herbal supplements, in rodent models, may be as effective as standard prescription drugs in managing anxiety (Bhattacharya and Muruganandam 2003). While our study did not directly evaluate the ability of Ashwagandha to cross the blood-brain barrier (BBB), existing literature suggests that the active compound of Ashwagandha, withanolide A, is capable of penetrating the BBB. Oral administration of Ashwagandha has been shown to be bioavailable, and its ability to cross the BBB likely contributes to its neuroprotective effects (Leonard et al. 2024; Mikulska et al. 2023). Moreover, it has been reported that Ashwagandha is considered a promising therapeutic agent for individuals with type 2 diabetes, mild cognitive impairment, and neurodegenerative diseases due to its ability to cross the BBB (Leonard et al. 2024).

CUMS in rodents is a commonly used model in depression studies due to its similarity to the physiology and pathology of human depression and involves exposing rodents to mild, unpredictable daily stressors (Willner 2017). This protocol is considered as a method close to modelling the human condition (Willner 2017; Wang et al. 2017). It has been shown that CUMS model applied for 15 days in Wistar albino rats is a powerful model for depression and anxiety, prevents habituation and activates inflammatory mechanisms (Blossom et al. 2020). In our study, it was shown that the 17-day CUMS procedure induced depressive-like behaviours. In adolescent rats exposed to the CUMS model of depression, pyknotic nuclei were observed in some glial cells and neurons with an increase in perivascular and perineural space, which may be a result of oedema accumulation in the cerebral structure. Moreover, in adolescent rats exposed to the CUMS model of depression, higher levels of pro-apoptotic Bax, caspase-3 and pro-inflammatory TNF-α and IL-1β, as well as decreased astrocyte-specific GFAP positive cell ratio and BDNF levels were associated with behavioural tests. These effects induced by the CUMS model were attenuated by Ashwagandha and sertraline treatments through at least partial inhibition of pro-apoptotic and neuro-inflammatory processes.

With the CUMS model, it is possible to establish specific aspects of depression symptomatology such as anhedonia, hopelessness and helplessness (Czéh et al. 2016; Willner 1997). Anhedonia, as one of the core symptoms of depressive disorder, is usually confirmed by a reduction in sucrose preference (Wiborg 2013). In our study, adolescent rats in the control group naturally showed a high sucrose preference, whereas adolescent rats induced with the CUMS model showed a significant decrease in sucrose preference. This was evaluated as a sign of anhedonia. Both 5.0 mg/kg/day sertraline and 50 mg/kg/day Ashwagandha treatments increased sucrose preference, suggesting that the treatments may have an ameliorative effect on depression-induced anhedonia.

In addition to anhedonia, the CUMS procedure triggers hopelessness/despair-like behaviour, another important feature of depression, and this can be assessed by FST (Aricioglu et al. 2019). Sertraline administration has been shown to decrease immobility and increase swimming behaviour in experimental animals and the results obtained are consistent with previous findings (Rogoz and Skuza 2006; Huang et al. 2019). In studies conducted in different depression models, Ashwagandha has also been shown to decrease the immobilisation time in FST (Hussein et al. 2021; Gupta and Rana 2007). In our study, adolescent rats exposed to CUMS remained immobile longer and swam less than healthy controls, confirming a hopelessness-like state. While sertraline and Ashwagandha treatments reversed these behaviours, no difference was found between the two treatments. These findings support the antidepressant-like effect of Ashwagandha.

The elevated maze test is used to test a rodent model of anxiety, but results from studies on the effects of SSRI treatment are conflicting, and it is still unclear whether these treatments are anxiety inducing or anxiety relieving (Borsini et al. 2002). In rats in which depression was induced by chronic mild stress model, it has been reported that entry into the open arm increased, the time spent in the closed arm was prolonged and anxiety-like behaviours were exhibited in the EPM test, and SSRIs may reduce these anxiolytic effects (Chiba et al. 2012). In our study, adolescent rats exposed to CUMS protocol, sertraline and Ashwagandha treated groups showed increased anxiety index compared to the control group. In all CUMS groups, rats showed an increase in closed arm duration and a decrease in open arm duration. Although sertraline and Ashwagandha treated groups showed a decrease in anxiety index in EPM compared to CUMS group, no significant difference was found.

In experimental animals subjected to chronic stress, it has been shown that stress causes depression-like symptoms such as anhedonia, sleep disturbance, loss of sexual interest, and weight changes (Haridas et al. 2013). It has been reported that weight gain decreases during stress in these animals, while weight gain increases during the recovery period (Chiba et al. 2012; Haridas et al. 2013). In our study, a significant increase in weight changes was observed between the CUMS group and the control group, and our findings are consistent with the literature. While weight gain decreased in the group treated with sertraline, weight gain in the group treated with ashwagandha was similar to the control group. These findings suggest that ashwagandha prevents weight loss associated with depression.

Oxidative stress and imbalance in redox homeostasis are considered to be an important pathological link in the development of depression together with neuroinflammation. Activation of microglia and astrocytes by oxidative stress leads to neuroinflammation (Shen et al. 2024). Inflammation has been shown to have an important place in the pathophysiology of depression due to the critical role of proinflammatory cytokines in regulating monoamine metabolism (Shen et al. 2024; Ma et al. 2022). Meta-analyses have shown that IL-1β and TNF-α levels are increased in depressed patients and are inversely proportional to antidepressant response (Hannestad et al. 2011; Dowlati et al. 2010). High levels of inflammatory mediators such as IL-1β and TNF-α in depression indicate that inflammation is an important factor contributing to the pathogenesis of depression (Shen et al. 2024). In addition, it has been reported that proinflammatory cytokines affect astrocytes, causing a decrease in glutamate reuptake and an increase in its release, which leads to a decrease in BDNF synthesis (Haroon and Miller 2017). BDNF plays a critical role in the development and neuronal remodeling of the central nervous system (Zhou at al. 2022). Chronic stress reduces BDNF levels, leading to hippocampal axon damage, cognitive impairment, and depression (Phillips 2017). Antidepressants such as SSRIs can increase BDNF levels, but the effects of sertraline on BDNF are controversial (Zhou at al. 2022; Talaee et al. 2024). The effects of Ashwagandha on stress have been investigated in clinical and preclinical studies in recent years. In clinical studies, decreased serum IL-1β and TNF-α levels after Ashwagandha intervention, suggesting that the stress-relieving effect of this plant may occur through antioxidant and anti-inflammatory mechanisms (Pandit et al. 2024). In animal models, Ashwagandha increased BDNF and GFAP expression and showed protective effects by reducing oxidative stress (Gautam et al. 2013; Konar et al. 2011). However, the effects of Ashwagandha on BDNF in adolescent depression have not been studied. In our study, TNF-α and IL-1β levels were significantly increased, while BDNF levels were significantly decreased in the CUMS group. In the depression group treated with sertraline, proinflammatory TNF-α and IL-1β levels were similar to the control and CUMS groups. In the Ashwagandha treatment, TNF-α levels were decreased compared to the CUMS group, but IL-1β levels were similar to the other groups. BDNF levels were similar between the depression and treatment groups. However, a borderline difference was found in BDNF levels between the CUMS and Ashwagandha treatments. Our results suggest that Ashwagandha may be a potential treatment approach for adolescent depression.

Dysregulation of NGF plays an important role in the pathophysiology of depression. Decreased NGF expression has been observed in rodent models (Mondal and Fatima 2019). Decreased NGF levels have been shown in postmortem hippocampal tissue of depressed and suicidal individuals. A study on platelets of rats reported that NGF release was reduced by sertraline (Hochstrasser et al. 2013). However, some studies show that NGF levels increase or remain unchanged after stress (Alleva et al. 1996; Lang et al. 2004). One study suggested that steroid lactones of Ashwagandha may activate TrkA by binding to the NGF binding site (Mitra et al. 2022). In our study, sertraline treatment did not change NGF levels, and NGF levels did not change in other groups.

Chronic stress has been shown to trigger neuronal apoptosis through increased capsase-3 activity and decreased anti-apoptotic proteins such as Bcl-2 (Orlovsky et al. 2014; Wang et al. 2020). Previous studies have shown that increased capsase-3 and Bax positive cells are present in the hippocampus of animal models exposed to chronic stress, and that upregulation of caspase activity via downregulation of neurotrophic factors such as BDNF promotes neuronal apoptosis (Dionisie et al. 2021). These effects increase neuronal cell damage and apoptosis, leading to the emergence of depressive behaviors (Lee et al. 2020). In our study, in the CUMS group, pro-apoptotic Bax and capsase-3 activities were increased compared to the control group, consistent with these findings. In the Sertraline-treated group, these pro-apoptotic activities were reduced and found to be at similar levels to the control groups. This finding supports the anti-apoptotic effects of Sertraline. Similarly, in the Ashwagandha-treated group, pro-apoptotic Bax and capsase-3 activities were significantly reduced compared to the CUMS group and found to be at similar levels to the control group. These results demonstrate the effects of Ashwagandha in reducing stress-induced apoptosis and its possible antidepressant effect. An important contribution of our study is that we examined these effects in adolescents. To the best of our knowledge, there are no studies in the literature comparing the effects of Ashwagandha in adolescent depression. Our findings suggest that Ashwagandha may have potential in preventing neuronal apoptosis in adolescents.

GFAP is one of the main structural proteins of astrocytes and is an important biomarker reflecting astrocyte function (Steinacker et al. 2021). One study reported that serum GFAP levels may help in the differential diagnosis of major depressive disorder and objectively measure depression severity, and may also be an effective marker for monitoring astrocyte pathology (Steinacker et al. 2021). Preclinical studies show that stress models reduce GFAP levels and that this effect can be reversed with antidepressant treatment (Araya-Callis et al. 2012; Banasr et al. 2010). These findings suggest that astrocyte pathology plays an important role in depression. The decrease in GFAP expression observed in the scopolamine-induced amnesia model was significantly restored by treatment with Ashwagandha (Wiborg 2013). Sertraline treatment reduced GFAP expression during neurogenic differentiation of human adipose tissue-derived stem cells (hADSCs), but did not show a significant change in the expression of the mature neuron marker (MAP2) (Razavi et al. 2014). Our study also supports these findings. In the CUMS group, the astrocyte-specific GFAP-positive cell ratio was significantly reduced compared to the control group. The GFAP-positive cell ratio in the Sertraline-treated group was similar to the control group. The GFAP-positive cell ratio in the Ashwagandha-treated group was similar to both the control and sertraline-treated depression groups. There was also a significant difference between the CUMS and Ashwagandha-treated depression groups. These findings suggest that the effects of stress on GFAP astrocytes can be reversed by both Sertraline and Ashwagandha treatment. Our study was conducted on adolescent individuals, and the lack of similar studies in this age group highlights the significance of our findings for this population.

We focused on the fronto-parietal cortex due to its role in cognitive and emotional processes. This region is involved in attention regulation, decision-making, and emotional control processes and is frequently impaired during depression (Wurm and Caramazza 2019; Schultz et al. 2018). A limitation of this study is that the exact cortical subregions analyzed in the fronto-parietal cortex were not specified. Instead, an analysis aimed to capture the overall activity of the fronto-parietal regions. This approach allowed us to understand the general stress response mechanisms in these regions. Although we focused on the fronto-parietal cortex as a broad region, detailed analyses on specific subregions (e.g., prelimbic, infralimbic, orbitofrontal cortex) may help us better understand the responses of these regions to stress and antidepressant treatment. Future studies should focus on specific cortical subregions to shed more light on the antidepressant-like effects of Ashwagandha. Another important limitation of our study is that only male adolescent rats were used, which makes it unclear whether there are gender differences in the antidepressant-like effects of Ashwagandha. Previous studies have suggested that there may be gender differences in both stress responses and antidepressant efficacy due to hormonal and neurobiological differences (Lewis et al. 2015; Wang et al. 2024). Estrogen and other gonadal hormones are known to modulate stress-related neural circuits, which may affect treatment outcomes. Future studies should investigate whether Ashwagandha exhibits similar antidepressant-like effects in adolescent female rats, as understanding gender-specific mechanisms is important for developing targeted treatment strategies. Investigating these differences will provide a more comprehensive view of the potential clinical significance of Ashwagandha in stress-induced depression.

## Conclusion

In adolescent rats subjected to the CUMS model, proapoptotic proteins such as Bax and Caspase-3, as well as inflammatory markers like TNF-α and IL-1β, were significantly increased compared to the control group, while the proportion of BDNF and GFAP immunopositive cells was decreased. These findings suggest that chronic stress impacts apoptosis and inflammation. While proapoptotic proteins and inflammatory markers were significantly elevated in the CUMS group, Ashwagandha treatment was more effective than Sertraline in reducing these levels. Notably, Ashwagandha treatment led to minor changes in BDNF levels and was effective in preventing weight loss. Furthermore, Ashwagandha reduced neuroinflammatory effects by positively altering glial markers. Ashwagandha demonstrated antidepressant-like effects on both cellular changes and neuroinflammatory processes, similar to Sertraline, in adolescent rats. Our findings suggest that Ashwagandha may have potential in improving adolescent depression, serving as a preliminary study in this area. This study represents an initial step toward understanding the potential antidepressant effects of Ashwagandha in adolescent depression and the underlying mechanisms involved. However, further research using additional animal models is needed to validate these results and establish a safe and effective treatment option.

## Data Availability

The data presented in this study are available on reasonable request from the corresponding author.
